# Nephrotic syndrome due to minimal-change disease superimposed on anti-glomerular basement membrane antibody positive glomerulonephritis; a case report

**DOI:** 10.1186/s12882-020-01947-x

**Published:** 2020-07-17

**Authors:** Yuko Shibata, Kazuhito Fukuoka, Riyo Yokota, Heryon Lee, Hikaru Sayo, Noriko Ikegaya, Kiyotaka Mori, Jin Yamamoto, Aya Isomura, Kiyotaka Nagahama, Hiroaki Shimoyamada, Takahisa Kawakami, Yoshinori Komagata, Shinya Kaname

**Affiliations:** 1 Department of Nephrology and Rheumatology, Kyorin University School of Medicine, 6-20-2, Mitaka-shi, Tokyo, 181-8611 Japan; 2 Department of Pathology, Kyorin University Hospital, Tokyo, Japan

**Keywords:** Ant-glomerular basement antibody glomerulonephritis (anti-GBM GN), Anti-glomerular basement antibody disease (anti-GBM disease), Minimal change nephrotic syndrome (MCNS), Plasma exchange, Intravenous cyclophosphamide, Atypical-anti-GBM glomerulonephritis

## Abstract

**Background:**

The prognosis for renal function in anti-GBM glomerulonephritis (anti-GBM GN) is extremely poor, and when renal impairment progresses severely, it is difficult to expect improvement. In addition, it is also known that once the disease activity can be controlled by aggressive treatment, its recurrence is rare. We experienced an anti-GBM GN that improved from severe renal dysfunction and relapsed. A possible cause was the superimpose of nephrotic syndrome due to minimal change disease (MCD).

**Case presentation:**

A 30-year-old man was admitted to our hospital because of general malaise, fever, oliguria and renal dysfunction. The patient’s laboratory data showed serum creatinine as high as 6.6 mg/dl, and severe inflammation (C-reactive protein 20.6 mg/dl). Anti-glomerular basement membrane antibody (anti-GBM Ab) was detected in his serum, which led to the diagnosis of anti-GBM GN. Treatment was initiated with high-dose glucocorticoid (GC) and plasma exchange therapy (PE), and the patient’s renal function and oliguria improved rapidly and he was discharged 40 days after admission. Renal biopsy findings showed cellular crescents associated with linear IgG depositions along the glomerular tufts compatible with anti-GBM GN, but only about one-third of the glomeruli was involved, suggesting that it still remains an early stage of the disease. However, 2 months after discharge, he had a relapse and was readmitted due to severe proteinuria with positive anti-GBM Ab. On the second admission, after high-dose GC and PE combined with intravenous cyclophosphamide, and remission was achieved. Despite the relatively minor renal biopsy findings, the patient showed rapid renal dysfunction and relatively rapid improvement with our treatment. Electron microscopy of the renal biopsy tissue showed significant foot process effacement on podocytes in the apparently normal glomeruli, without electron dense deposits.

**Conclusion:**

On the basis of clinical course and renal pathology, it is suggested that the present case was a rare complication of an early stage of anti-GBM GN and minimal change nephrotic syndrome. Although the simultaneous development of anti-GBM GN and MCD with anti-GBM antibody is unclear, it might have been precipitated by influenza infection or some unknown factor.

## Background

Anti-glomerular basement membrane (GBM) antibodies are autoantibodies to the non-collagenou-1(NC-1) domain of type IV collagen and are found as a disease-specific marker for Goodpasture syndrome (GS) [1]. GS is currently classified as an anti-GBM disease in the nomenclature of the Revised International Chapel Hill Consensus Conference 2012 to systemic vasculitis as an immune-complex small vessel vasculitis [2]. Especially when it is confined to renal lesions, the term anti-GBM glomerulonephritis “(anti-GBM GN)” is widely used [3]. Anti-GBM GN is the typical presentation of rapidly progressive glomerulonephritis (RPGN), and its prognosis is extremely poor. It is well known that most patients with a glomerular filtration rate (GFR) of less than 15 ml/min will be on maintenance dialysis. In addition, anti-GBM GN has also known as “one-hit phenomenon” and its recurrence is also extremely rare [3]. A few reports have shown the involvement of exogeneous factors such as cytomegalovirus, carbon tetrachloride, and smoking [4] in its recurrence [5–7]. In other words, it may be suggested that anti-GBM GN rarely recurs unless there is a specific exogenous factor exposure or a specific genetic background.

Here we experienced a case of anti-GBM GN with heavy proteinuria and severe renal dysfunction, which improved markedly and relapsed 2 month later. The treatment was successful and the recurrence also promptly improved. In our case, nephrotic syndrome due to minimal-change disease (MCD) was thought to superimposed on anti-GBM GN.

## Case presentation

**Fig. 1:**
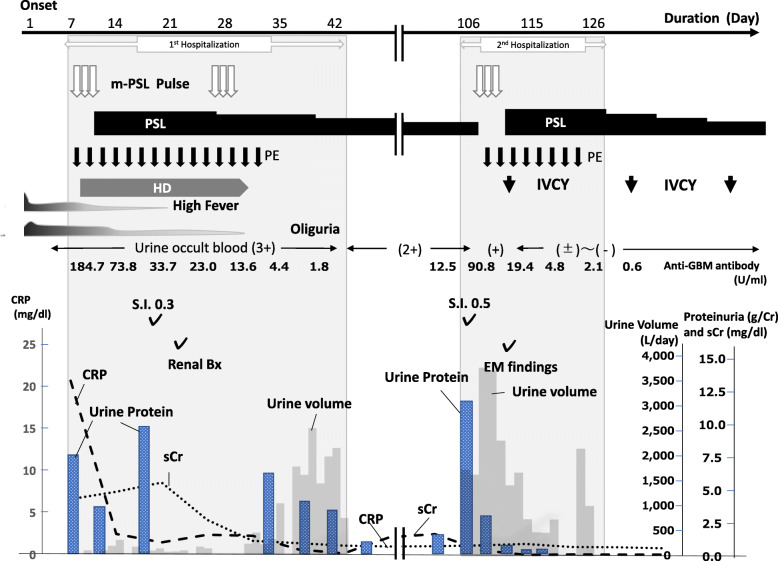
Clinical course of present case. The patient was admitted to our hospital with high fever and oliguria. At the time of his visit, he was accompanied by a markedly elevated serum creatinine and was immediately started on GC pulse and PE therapy. He also underwent concomitant hemodialysis for severe renal impairment. Subsequently, high levels of anti-GBM antibody was detected and anti-GBM GN was diagnosed. With these treatments, his renal function quickly improved, proteinuria and serum GBM antibodies became negative, and he was discharged on the 46th day of hospitalization. However, 2 months later, he had a relapse with positive anti-GBM antibody and increased proteinuria. He was admitted to our hospital again and received GC pulse and PE, plus IVCY therapy. As a result, the patient was again in remission and discharged from our hospital. sCr: serum creatinine, GC: glucocorticoid, PE: plasma exchange therapy, HD: hemodialysis, S.I: selectivity index, EM: electro-microscopy, m-PSL: methyl-prednisolone, GBM: glomerular basement membrane antibody, CRP: c-reactive protein, anti-GBM GN: anti-glomerular basement membrane glomerulonephritis, IVCY: intravenous cyclophosphamide

A 30-year old man came to our hospital due to a pyrexia, oliguria and general malaise that persisted for 10 days. He suddenly presented with a high fever and general malaise 10 days before and was transferred to our hospital because elevated leukocyte counts, a marked inflammatory reaction and a decline in kidney function were shown in the previous clinic (Fig. 1). He had been unable to eat or drink for more than a week due to severe appetite loss. He had a past clinical history of ulcerative colitis (UC) at the age of 23, but had been in remission for the past few years and had taken no medication for UC anymore. In addition, he was a current smoker and had habitual drinking, he also has a family history of rheumatoid arthritis in his mother. On admission, the patient’s blood pressure was 130/70 mmHg, pulse rate was 70/min regularly. Body temperature had risen to 38.8 °C. His body weighed 70.4 kg, despite not getting enough food and drink for nearly a month, his weight increased of 400 g from the previous months. Physical examination revealed high fever on admission, but there were no other abnormal findings including pitting edema on his limbs. The blood test showed with a moderate anemia of hemoglobin 10.5 g/dl, and a leukocytosis with 11,400/μl (neutrophil dominant). He developed severe renal impairment, with an increase of blood urea nitrogen (BUN) and serum creatinine (Cr), 36.1 mg/dl and 6.68 mg/dl respectively. An elevated inflammatory response was noted (c-reactive protein 20.75 mg/dl) and serum procalcitonin level was elevated, but there were no obvious signs and symptoms of infection. Serum albumin was found to have decrease to 1.2 g/dl. Anti-GBM antibody was high at 184.7 U/l, but both anti-myeloperoxidase and protease 3 neutrophil cytoplasmic antibody (ANCA) were negative. Although he presented oliguria (< 400 ml), urine findings showed a large number of glomerular erythrocytes and diverse sediments; numerous urinary RBCs and granular cast (with high power field), and 50–99 of epithelial and waxy casts were observed (with wide field), and the results of urine biochemistry and electrolytes were as follows; protein 702 mg/dl, 5.28 was found as urine protein estimated by gram creatinine (gCr), Na 12 mmol/L, UN 244 mg/dl, Cr 133 mg/dl, N-acetyl-β-D-glucosaminidase (NAG) 32.1 IU/L, and β2-microglobulin 3720 μg/l. Fractional excretion of sodium (%FEna) and of urea nitrate (%FEun) were 0.4 and was 5.57 respectively, and selectivity index (S.I.) calculated by urine IgG and transferrin was 0.30. There were no findings of cardiac enlargement and alveolar hemorrhage on the chest x-ray. Abdominal echocardiography and abdominal computed tomography showed bilateral renal swelling and no other noteworthy findings.

**Fig. 2:**
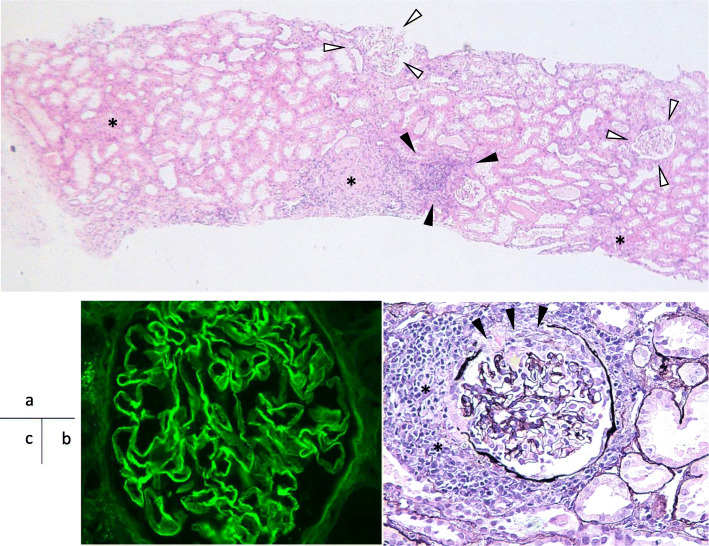
Microscopic findings of kidney biopsy (**a**) 100x PAS staining. Globally inflamed glomeruli (arrows) and cell infiltration into the interstitium (asterisks). The lesions were distributed focally, and impaired glomeruli were found in approximately one-third of the total. Less impaired glomeruli were also scattered (open arrow). (**b**) 400x PAS staining. Cellular crescent with ruptured Bowman’s capsule (arrows). It was also seen a glomerular capillary gap (yellow asterisk). There was significant cellular infiltration around the Bowman’s capsule (asterisks). (**c**) Immunofluorescent antibody staining (IgG). IgG was stained along the glomerular capillary wall in a linear pattern, suggesting the deposition of anti-GBM antibodies. PAS stain: Periodic acid-Schiff stain

**Fig. 3:**
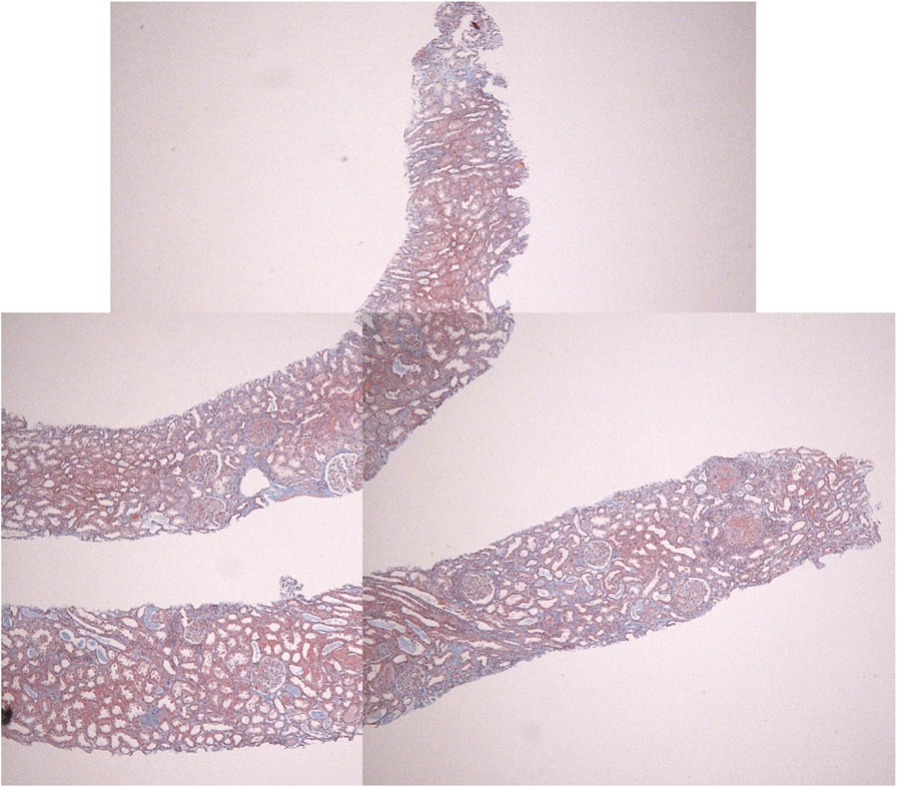
Masson staining 100x. Fibrotic change of tubular interstitium was mild, and observed in about 10% of specimen. In addition, there was little or no edema of the tubular interstitium

After hospitalization, because anti-GBM GN was highly suspected, we decided to begin the treatment with plasma exchange (PE) and methyl-prednisolone pulse therapy followed by 60 mg of prednisolone. In addition, since the serum Cr was already high (7.62 mg/dl) and presented an oliguria, hemodialysis was also begun from the day 5th of hospitalization. A renal biopsy was performed on the day 13th of hospitalization. The pathological findings of renal biopsy showed necrotic changes with rupture of the basement membrane, significant cellular crescentic formation complicated with severe interstitial cell infiltration, and glomeruli with global sclerosis, but these affected lesions distributed as focal, and accounted for only one-third of approximately 30 glomeruli observed, and the remaining glomeruli were surprisingly showed minimal change (Fig. 2 a and b). The specimen stained with fluorescent antibody showed a linear deposition of IgG along the glomerular capillary wall (Fig. 1c). Although there was a poor response to the initial glucocorticoid (GC) pulse, renal biopsy findings showed a small number of glomeruli that had fallen into sclerosis, suggesting the possibility of improvement. Masson staining showed about 10% fibrosis of the tubular interstitium and little or no interstitial edema (Fig. 3). Therefore, PE was continued after the kidney biopsy and another GC pulse therapy was also added. We also discussed the use of intravenous cyclophosphamide therapy (IVCY). However, given his severe renal dysfunction and oliguria, we decided to postpone IVCY until his renal function improved, as it was feared he would complicate severe side effects such as bone marrow suppression. After that, the anti-GBM Ab became negative and the renal function improved significantly, and proteinuria decreased, and he was withdrawn from hemodialysis on the 26th day after admission. On the 40th day after admission, he was able to be discharged from our hospital. At the time of discharge, proteinuria was 0.4 g/day and serum Cr level declined to 1.15 mg/dl. The addition of the IVCY was not done because of the remaining mild renal dysfunction and also because the patient had become negative for anti-GBM antibody and relapses were thought to be occured rarely in this disease.

**Fig. 4:**
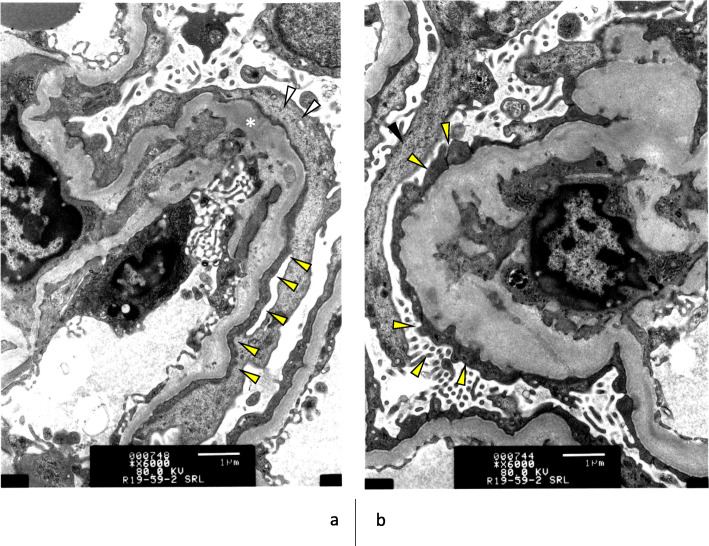
electron microscopic findings (**a**) and(**b**) Glomerular basement membrane with deposits (asterisk), and was covered by foot process fusions of podocytes (yellow arrow). Cell body of podocyte are indicated by white arrow

Two months after discharge, the anti-GBM Ab became positive again (90.8 U/ml), and the nephrotic syndrome recurred, and he was readmitted to our hospital. On the second admission, he had systemic edema and showed severe proteinuria (16.0 g/day), but serum Cr level was 1.0 mg/dl. Serum albumin was 2.6 g/dl, and CRP was 2.5 mg/dl. Again, GC pulses and PE were started. In addition to these, we planned to administer 750 mg of IVCY for 6 months, and on the fifth day of second hospitalization, the first administration has begun. Around that time, the results of a renal biopsy analyzed by electron microscopy (EM) arrived, and the renal pathology was reviewed (Fig. 4). In the glomeruli which were not shown sclerotic or necrotic change in EM, there were remarkable effacements of podocyte foot process. Considering the clinical course and pathological findings, he was diagnosed as a superimpose of nephrotic syndrome due to minimal change disease (MCD). Finally, the anti-GBM antibody turned negative, and the proteinuria quickly disappeared (0.13 mg/day), so he was discharged from our hospital on the 21st day of second admission. At the end of this case presentation, we have explained to him in detail about his condition gave and future course of treatment and examinations in each case, and have treated him with his consent.

## Discussion and conclusion

We experienced a case of anti-GBM GN with possible MCD. Although the patient had severe renal failure at the time of visiting our hospital, he showed marked improvement after aggressive immunosuppression therapy combined with PE.

Anti-GBM GN and anti-GBM disease are sometimes associated with other nephritis. The association of ANCA-associated nephritis with anti-GBM GN has been reported to occur in approximately 12.8% of cases [8]. It is also known that membranous nephropathy (MN) is associated with anti-GBM GN [9]. Anti-GBM GN complicated with MCD is extremely rare, to our knowledge, with only two cases in the literature, including the conference proceedings [10, 11]. A complication of MCD might be unlikely to be found because anti-GBM GN progresses so rapidly that the patients in many cases are already in end-stage renal failure at the time of diagnosis and also the anti-GBM antibody GN can sometimes cause heavy proteinuria in the range of nephrotic syndrome. There are two reasons why we considered this case may be a complication of anti-GBM GN and MCD.

First, this case recovered from severe renal impairment. The untreated renal prognosis in anti-GBM disease was only 2% and the life expectancy was also remarkably poor (4%) [4]. Segelmark et al. analyzed a total of 12 (659 cases) case series of previously reported anti-GBM GN from 1975 to 2015 and described that the life prognosis has improved in recent years however, the renal survival rate remains low (approximately 26%), and almost difficult to avoid hemodialysis, especially in cases with delayed diagnosis, GFR below 15 ml/min, or anuria at the time of diagnosis [12]. Levy et al. reported the watershed of recovering is about 5.7 mg/dl of serum creatinine level [4]. Our case already had a serum creatinine of 6.6 mg/dl and an eGFR of only 9.3 ml/min at the time of first admission. In general, it is highly unlikely that maintenance dialysis will be avoided when renal function is so compromised by anti-GBM GN. While in MCD, pre-renal acute kidney injury (AKI) is often associated, and it is sometimes shown for patients who needed to be on dialysis for a short period of time to subsequently be free of maintenance dialysis with GC treatment. If the initial advanced renal failure was pre-renal AKI due to concomitant MCD rather than RPGN due to anti-GBM GN, high-dose GC may improve and explain this condition. In addition, the patient had severe appetite loss for more than a week and also had a high fever, which thought to be also contributed to worsen dehydration and led to the development of AKI earlier periods. The second rationale is renal pathology findings. In our case, only about 1/3 of the glomeruli were damaged, and the distributed in a focal pattern, which may have been early or mild for anti-GBM GN. In anti-GBM GN, the rate of crescent formation is the most useful predictor of renal prognosis, with a crescent formation rate of more than 85% reportedly associated with extremely poor improvement in renal function [4, 13–15]. In spite of these findings, proteinuria was extremely high. In addition, a large number of effacement of podocyte foot process were observed in glomeruli that were less impaired by EM. Because the pathogenesis of anti-GBM GN is impaired by glomerular basement membrane antibodies, it is inconsistent that podocyte effacement in unimpaired capillaries. About the renal pathology findings not showing severe interstitial edema, it suggested that his AKI was not only caused by the under-filling mechanism of nephrotic syndrome but also severe dehydration. Taken together, we found that in our case, anti-GBM GN was superimposed by MCD.

Given the response to treatment and pathological findings shown in our case, it is necessary to consider about atypical GBM glomerulonephritis (atypical GBM GN) as a different diagnosis. Atypical GBM GN was described by Jennette et al. in 2003 as a group with milder or moderate tissue lesions than conventional anti-GBM GN, and with no typical RPGN and less severe clinical presentations despite the presence of GBM antibody deposition in the GBM [16]. This Atypical GBM GN often has a better renal function prognosis than the classic GBM GN, but it often presents with proteinuria and often reaches the nephrotic range [17]. In addition, pulmonary lesions are rarely complicated and renal recurrence is occasionally reported. Considering the clinical course and the pathological findings had a lot in common in our case. It seems difficult to rule out the possibility that our case was also atypical GN. However, in our case, serum anti-GBM antibody level was high at the onset of the disease (197 U/L), negative after treatment-induced remission, and increased at relapse (90 U/L), suggesting that serum anti-GBM Ab levels well reflected disease activity. From several studies, an indolent clinical course and pathological findings of atypical GBM have been attributed to the assuming that antibodies in atypical GBM GN recognize a different epitope from α3NC1, unlike GBM Ab in conventional anti-GBM GN, therefore, in atypical anti-GBM GN, serum anti-GBM antibodies is usually unproven or very low and there seems to be little correlation between antibody titers in sera and the disease activity. In addition, in our case, renal biopsy showed only IgG deposition without any other immunoglobulins, and moreover, there was a rupture of Bowman’s capsule, without proliferation of mesangial and endothelial cells and membranous lesion that are usually observed in atypical anti-GBM GN. Furthermore, impaired lesion was not large enough to be able to cause severe proteinuria leading to pre-renal AKI. In view of these, we considered the most reasonable diagnosis to be that “typical GBM GN “was superimposed by MCD and that pre-renal renal failure due to nephrotic syndrome led to early detection and successful treatment.

The mechanism of direct association between anti-GBM GN and MCD is unknown, but may include the presence of self-reactive T cells. Although it is difficult to find out a common pathway, it seems reasonable to assume that a prior infection caused both at the same time. Anti-GBM disease is known to develop after influenza infection [18]. It has been suggested that lung damage triggered by prior infection can produce autoantibodies that may react to the antigen of GBM. At the same time, there have been a few reports of MCD in children after influenza infection and a few reports of MCD after influenza vaccination [18–21]. Our case had a high fever 10 days prior to onset and was suspected of having an influenza infection. The low likelihood of recurrence of anti-GBM GN and what is known as the “one-hit phenomenon” [3]. In general, anti-GBM disease, autoantibody titers, and auto-reactive T-cell numbers have decreased after discontinuation of immunosuppressive drugs, suggesting that the reason for this is a restored immune tolerance to α(IV)NC1 [22, 23]. Despite that recurrence is extremely rare, in our case, it occurred 2 months after discharge. Assuming that the onset was triggered by an influenza infection 3 month before, the time to relapse may have been short and the possibility either the number of autoreactive T cells was not sufficiently reduced, or the acquisition of self-tolerance was inadequate, cannot be ruled out. Meanwhile relapses in MCD are more frequent and often occur during GC reduction. Recent findings have also shown that when treated with steroids alone, the recurrence rate can be as high as 60% in 3 months [24]. Taken together, it could be speculated that a response to prior infection such as influenza caused the development of GBM nephritis and MCD simultaneously. At the time of the recurrence, the patient was positive for anti-GBM antibodies but had increased proteinuria and systemic edema rather than worsening renal function.

Finally, we would like to add some commentaries about the course of treatment. KDIGO guidelines have shown a good prognosis for treatment concomitant with IVCY from early phase [25, 26]. In spite that, in our case, we did not use IVCY from the beginning. The reason for this was that our patient was initially considered to have anti-GBM nephritis alone, and thereby was considered to be not only unresponsive to treatment but also high-risk due to the already high serum Cr level at the time of admission. However, based on the good response to treatment and the results of the renal biopsy, strongly suggested to have been complicated with MCD. Therefore, on the second admission, we added an IVCY. Of course, high-dose GC therapy for MCD and cyclophosphamide have been shown to be effective in maintaining remission, especially in relapsing patients [27, 28]. As a result, these treatments successfully led him to a remission.

In conclusion, we experienced an anti-GBM antibody positive severe renal impairment complicated by MCNS. Although it is unknown whether this is just a coincidence or not, it reminds us of the importance of performing kidney biopsy to determine accurate diagnosis, treatment plan and prognosis for anti-GBM positive patients even in patients with severe renal impairment.

## Data Availability

All data generated or analyzed data were obtained from Kyorin University Hospital, and are included in this published article.

## Abbreviations

NC-1: non-collagenous lesion 1
GS: Goodpasture syndrome
GBM: glomerular basement membrane
anti-GBM Ab: anti-GBM antibody
anti-GBM GN: anti-GBM glomerulonephritis
RPGN: rapidly progressive glomerulonephritis
BUN: blood urea nitrogen
gCr: gram creatinine as calculated by urine protein creatinine ratio
Cr: creatinine
GFR: glomerular filtration rate
ANCA: anti-neutrophil cytoplasmic antibody
IVCY: intravenously administration of cyclophosphamide
MCD: nephrotic syndrome disease
GC: glucocorticoid
PE: plasma exchange
AKI: acute kidney injury
